# Job satisfaction and absenteeism among Brazilian teachers

**DOI:** 10.47626/1679-4435-2023-1054

**Published:** 2023-11-24

**Authors:** Adrieli de Fátima Massaro Levorato, Selma Maffei de Andrade, Giovana Frazon de Andrade, Edmarlon Girotto

**Affiliations:** 1 Instituto Adrieli Massaro, Londrina, PR, Brazil; 2 Programa de Pós-Graduação em Saúde Coletiva, Universidade Estadual de Londrina (UEL), Londrina, PR, Brazil; 3 Universidade Estadual do Centro Oeste, Guarapuava, PR, Brazil; 4 Programa de Pós-Graduação em Saúde Coletiva, Programa de Pós-Graduação em Ciências Farmacêuticas, UEL, Londrina, PR, Brazil

**Keywords:** absenteeism, school teachers, job satisfaction, absenteísmo, professores escolares, satisfação no emprego

## Abstract

**Introduction:**

Teachers, especially those in primary education, face unfavorable working conditions, which lead to job dissatisfaction and affect their physical and mental health, thus contributing to absenteeism.

**Objectives:**

To verify the association between lower job satisfaction and absenteeism due to short and long term health problems in elementary and hight school teachers.

**Methods:**

This observational, analytical, individual, cross-sectional, retrospective cohort study included 899 elementary and high school teachers. Absenteeism was determined by self-reported absences in the last 12 months for health reasons, categorized as short term (1-7 days) or long term (≥8 days). Job satisfaction was measured by the Occupational Stress Indicator scale, categorized as lower satisfaction (≤25th percentile) or higher satisfaction (>25th percentile). Multinomial logistic regression was used, and the odds ratio was calculated as a measure of association.

**Results:**

The majority of the teachers were women (68.3%) and were permanently employed (69.1%); the mean age was 42 (SD, 10) years. Women, younger teachers, permanent employees, those reporting chronic pain or illness, and those reporting a moderate/poor level of physical or mental work capacity had a higher risk of absenteeism. Lower job dissatisfaction was associated with short-term and long-term absenteeism. Job satisfaction was only related to short-term absenteeism after the adjustments made.

**Conclusions:**

There was an association between absenteeism and lower job satisfaction, which indicates that measures to improve job satisfaction are necessary.

## Abstract (pt)

**Introdução:**

Professores, especialmente os da educação básica, têm enfrentado inúmeras condições desfavoráveis de trabalho, o que gera insatisfação no trabalho, com prejuízo à sua saúde física e mental, contribuindo para o absenteísmo.

**Objetivos:**

Verificar a associação entre menor satisfação no trabalho e absenteísmo por problema de saúde de curto e longo prazos em professores do ensino fundamental e médio.

**Métodos:**

Estudo observacional, analítico, individual, transversal, retrospectivo de coorte com professores do ensino fundamental e médio (n = 899). O absenteísmo foi determinado pelo relato de falta nos últimos 12 meses por motivo de saúde, categorizado em curto prazo (1 a 7 dias) e longo prazo (8 dias ou mais). A satisfação no trabalho foi mensurada pela escala Occupational Stress Indicator, categorizada em menor satisfação (até o percentil 25) e maior satisfação (acima do percentil 25). Foi utilizada a regressão logística multinomial, com cálculo da *odds ratio* como medida de associação.

**Resultados:**

Dos professores avaliados, a maioria era do sexo feminino (68,3%), tinham média de idade de 42 ± 10 anos e apresentavam vínculo estatutário (69,1%). As chances de absenteísmo foram maiores entre os professores do sexo feminino, mais jovens, com vínculo estatutário, que referiram dor ou doença crônica e que relataram capacidade física ou mental para o trabalho moderada/baixa. Professores menos satisfeitos apresentaram maiores chances de absenteísmo de curto e de longo prazo. Após os ajustes realizados, a satisfação no trabalho associou-se apenas ao absenteísmo de curto prazo.

**Conclusões:**

Houve associação entre absenteísmo e menor satisfação no trabalho, tornando-se necessárias medidas para melhorar a satisfação no trabalho.

## INTRODUCTION

The work of teachers consists of numerous academic and administrative activities beyond the act of teaching, given that it involves planning and preparation, developing assessments, filling out numerous forms, and participating in meetings, many of which occur outside working hours.^1^ Such overload can lead to health problems, such as musculoskeletal pain and psychological disorders.^2,3^

In Brazil, the precariousness of the public school system further aggravates job satisfaction.^4,5^ Job dissatisfaction is related to the everyday problems and challenges of work, such as low pay, school violence, and inadequate infrastructure, among other factors.^6,7^ These aspects affect individuals according to their beliefs, values, and personal perspective, with compromised work activity reflected in physical and mental health disorders^8^ and absenteeism.^9,10^

Absenteeism, defined as an employee’s failure to show up for work, is a multicausal phenomenon determined by factors such as health conditions, the work environment, and the nature of the work activity.^11,12^ Since this problem has proven recurrent among primary and secondary school teachers and may result in setbacks to the educational process,^13^ it is important to better investigate its determinants.

Several predictors of absenteeism among workers in different professions have been identified, such as musculoskeletal pain and chronic diseases,^14^ workload, work intensity, physical and mental fatigue, in addition to sociodemographic factors such as marital status, age, and sex.^13,15^ Among Brazilian public school teachers, these factors have shown a greater association with absenteeism, especially in light of the stress and emotional problems teachers report.^7^

Investigations into the relationship between job satisfaction and absenteeism are little investigated, and studies on this association have mainly focused on health professionals^9,10,16,17^ or workers in general;^18,19^ no studies on this topic could be found regarding public school teachers. Absenteeism is a multifactorial problem, and further investigation of other causal aspects, such as job satisfaction, could lead to different views and perspectives on the scope of the problem, in addition to identifying the conditions to which teachers are exposed. Therefore, this study’s objective was to verify the association between lower job satisfaction and shortand long-term absenteeism due to health problems among primary and secondary public school teachers, regardless of the health condition or demographic or socioeconomic factors.

## METHODS

### STUDY DESIGN

This cross-sectional study is part of a research project entitled “*Saúde, Estilo de Vida e Trabalho de Professores da Rede Pública do Paraná* (Pró-Mestre)” [The Health, Lifestyle, and Work of Public School Teachers in Paraná]. All information from this study is presented in accordance with STROBE (Strengthening the Reporting of Observational Studies in Epidemiology) guidelines.^20^

### STUDY LOCATION AND POPULATION

The study population comprised all of the teachers working in the 20 regular public primary and secondary schools in the city of Londrina, the largest number of any municipality in the Paraná state education network. These schools were intentionally selected to facilitate logistics, in addition to the fact that approximately 70% of Londrina’s primary and secondary school teachers work in them. A total of 1251 working teachers were identified in these schools through the Londrina Regional Education Center.

This study included teachers with at least 1 year of experience. Teachers who were readapted or in the readaptation process (ie, those whose health conditions that permanently prevent them from teaching) were excluded. Teachers who had been away from work for ≥180 days in the 12 months prior to data collection were also excluded, due to limited work exposure. There was no sampling process, since all teachers from the selected schools were approached if they met the inclusion criteria.

### DATA COLLECTION

A pilot study was conducted with 82 teachers in 3 schools in a neighboring municipality to assess the study procedures and test the data collection instruments.

Data were collected between August 2012 and June 2013 in a designated space in the schools when the teachers were not in class. Trained researchers, who received a support manual for the data collection instrument, applied an interview of approximately 40 minutes, after which the participants answered a self-administered questionnaire, which took approximately 10 minutes. The self-administered questionnaire was a way to obtain answers to more sensitive questions, such as monthly family income, and to apply health scales for which self-responses are recommended. All interviews were conducted in a private environment to ensure confidentiality.

Of the 1251 teachers selected for the study, 138 were excluded (125 due to readaptation, 9 due to having worked <1 year, and 4 for reporting an absence >180 days in the previous 12 months). Thus, 1113 teachers were considered eligible for the study. Of these, 214 were considered losses (65 were on leave, 63 refused, 20 could not be located, 50 did not fully complete the job satisfaction scale, and 16 did not respond or responded inconsistently to questions about absenteeism). Therefore, the final sample consisted of 899 teachers (80.8% of the eligible teachers).

### STUDY VARIABLES

Absenteeism, the dependent variable of this study, was determined as follows: all teachers who reported having been absent in the last 12 months for health reasons were asked about the number of full days they missed from work. Absenteeism was then classified as short-term (1-7 days) or long-term (≥8 days). Maternity/paternity leave and absences due to routine prenatal consultations or procedures were not considered.

Job satisfaction, the independent variable, was measured using the Occupational Stress Indicator job satisfaction scale,^21^ which was translated to Brazilian Portuguese and validated by Swan et al..^22^ This scale consists of 22 items on aspects of job satisfaction (eg, salary, workload, and relationships), which are rated according to satisfaction level (immensely satisfied, very satisfied, some satisfaction, some dissatisfaction, very dissatisfied, and immensely dissatisfied). The sum of responses can range from 22 (lowest satisfaction) to 132 (highest satisfaction). Since there is no validated cutoff point for this scale, we selected the 25th percentile to distinguish lower satisfaction (≤25th percentile) from higher satisfaction (>25th percentile).

The adjustment variables were demographic and socioeconomic: sex (male or female) age, in tertiles (23-35 years, 36-46 years, or 47-68 years); marital status (married/cohabiting or single); employment contract type (temporary or permanent); monthly family income (≤ BRL 3000.00 or > BRL 3000.00), and variables related to health conditions (reported chronic pain [yes or no]; chronic disease[yes or no]; perceived physical work capacity (very good/good or moderate/low); and perceived mental work capacity (very good/good or moderate/low). Chronic pain was defined as frequent pain in one or more body regions lasting ≥6 months.^23^ The following diseases were considered chronic: high blood pressure, diabetes, depression, anxiety, arthritis, osteoarthritis, rheumatism, asthma, emphysema, bronchitis, malignant tumor, heart failure, pulmonary tuberculosis, and kidney disease.

### STATISTICAL METHODS

The data obtained through the collection instruments were checked by field coordinators and then double entered. The two files were compared in Epi Info 3.5.4 and any discrepancies were corrected. The data were then tabulated in IBM SPSS Statistics 19.0.

A descriptive analysis was performed using absolute and relative frequencies for qualitative variables and measures of central tendency and dispersion for quantitative variables. The reliability of the job satisfaction scale was also analyzed using Cronbach’s alpha coefficient. Multinomial logistic regression was used for bivariate and multivariate analyses; the odds ratio (OR) with respective 95%CI was calculated as a measure of association. The following categories were considered in analyses for absenteeism (dependent variable): 0 days (reference), 1-7 days (short term), and ≥8 days (long term). Furthermore, the model’s confounder matrix was assessed to determine its sensitivity and the percentage of correctly classified cases.

Two adjusted models were constructed to analyze the relationship between job satisfaction and absenteeism, including sex, age, and variables with p < 0.20 in the unadjusted analysis. Model 1 included were socioeconomic and demographic variables, while Model 2 included the variables from Model 1 plus those related to health conditions.

### ETHICAL ASPECTS

The *Pró-Mestre* project was approved by the State University of Londrina Research Ethics Committee (CAAE 01817412.9.0000.5231). The research objectives were explained to the participants and, after they provided written informed consent, data collection began.

## RESULTS

The mean age of the 899 teachers was 42 (SD, 10) years (range 23-68). The mean time in the profession was 14 (SD, 9) years, varying between 1 and 45 years. The majority of teachers had a permanent employment contract (69.1%). Just under half of the teachers reported chronic pain (42.8%), and a large majority reported some chronic disease (84%).

The mean score on the job satisfaction scale was 84 points (median 85; range 26-131). The cutoff point for lower job satisfaction was 72 points (25th percentile); this group included 229 teachers. The Cronbach’s alpha coefficient for job satisfaction was 0.943.

The items with the lowest satisfaction levels (great or significant dissatisfaction) were salary (46.9%), work volume (29.6%), and opportunities to achieve aspirations and ambitions (21.5%).

A total of 453 (50.4%) teachers reported having missed work due to health problems in the previous 12 months: 31.5% for 1-7 days (short-term) and 18.9% for ≥8 days (long term). The mean number of full days missed was 7.5 (SD, 27.3; median 5.0; range 1-180). Other characteristics of the participants are shown in Table 1.

**Table 1:** Demographic and socioeconomic characteristics of public school teachers in Londrina, PR, Brazil, 2012-2013

Variables	Total n (%)
Sex	
Male	285 (31.7)
Female	614 (68.3)
Age (years)	
23-35	288 (32.0)
36-46	309 (34.4)
47-68	302 (33.6)
Marital status^*^	
Married/cohabiting	533 (59.3)
Single	364 (40.6)
Contract type	
Temporary	278 (30.9)
Permanent	621 (69.1)
Family income^*^ (BRL)	
≤ 3000.00	222 (24.7)
> 3000.00	675 (75.3)
Chronic pain reported^*^	
Yes	384 (42.8)
No	513 (57.2)
Chronic disease reported	
Yes	755 (84.0)
No	144 (16.0)
Physical work capacity^*^	
Very good/good	545 (60.7)
Moderate/low	353 (39.3)
Mental work capacity^*^	
Very good/good	631 (70.3)
Moderate/low	267 (29.7)

* The total number of participants was lower due to missing information in the database.

Female sex and permanent contract type were associated with long-term absenteeism. Age 23-35 years was associated with both shortand long-term absenteeism. Age 36-46 was only associated with short-term absenteeism. There was also an association between chronic pain, chronic illness, and moderate/low perceived mental capacity for work and both shortand long-term absenteeism. Moderate/low perceived physical work capacity was only associated with long-term absenteeism (Table 2).

**Table 2:** Association between demographic and socioeconomic characteristics and absenteeism in public school teachers, Londrina, PR, Brazil, 2012-2013

Variable	Short term (n = 283)			Long term (n = 170)		
	n (%)	OR (95% CI)	p-value	n (%)	OR (95% CI)	p-value
Sex						
Male	94 (33.0)	1.00		39 (13.7)	1.00	
Female	189 (30.8)	1.04 (0.76-1.43)	0.810	131 (21.3)	1.74 (1.16-2.61)	0.008
Age (years)						
23-35	105 (36.5)	1.65 (1.14-2.41)	0.009	43 (14.9)	0.73 (0.47-1.13)	0.160
36-46	105 (34.0)	1.60 (1.10-2.32)	0.014	59 (19.1)	0.96 (0.64-1.46)	0.860
47-68	73 (24.2)	1.00		68 (22.5)	1.00	
Marital status^*^						
Married/ cohabiting	171 (32.1)	1.08 (0.79-1.46)	0.610	101 (18.9)	1.05 (0.73-1.50)	0.841
Single	112 (30.8)	1.00		68 (18.7)	1.00	
Contract type						
Temporary	98 (35.3)	1.00		34 (12.2)	1.00	
Permanent	185 (29.8)	0.92 (0.67-1.26)	0.598	136 (21.9)	1.95 (1.27-2.98)	0.002
Family income^*^ (BRL)						
≤3.000.00	72 (32.4)	1.02 (0.73-1.44)	0.867	39 (17.6)	0.89 (0.59-1.36)	0.615
>3.000.00	211 (31.3)	1.00		131 (19.4)	1.00	
Chronic pain reported^*^						
Yes	125 (32.6)	1.36 (1.01-1.84)	0.049	94 (24.5)	2.13 (1.49-3.08)	< 0.001
No	157 (30.6)	1.00		75 (14.6)	1.00	
Chronic disease reported						
Yes	247 (32.7)	1.98 (1.31-3.00)	0.001	162 (21.5)	5.85 (2.78-12.32)	< 0.001
No	36 (25.0)	1.00		8 (5.6)	1.00	
Physical work capacity^*^						
Very good/good	169 (31.0)	1.00		81 (14.9)	1.00	
Moderate/low	113 (32.0)	1.31 (0.96-1.78)	0.090	89 (25.2)	2.15 (1.50-3.07)	< 0.001
Mental work capacity^*^						
Very good/good	186 (29.5)	1.00		108 (17.1)	1.00	
Moderate/low	96 (36.0)	1.60 (1.15-2.21)	0.005	62 (23.2)	1.78 (1.21-2.60)	0.003

OR = *odds ratio*.

* The total number of participants was lower due to missing information in the database.

† p < 0.05.

‡ p < 0.01.

§ p < 0.001.

Teachers with lower job satisfaction had a higher risk of short-term (OR 1.89; 95% CI 1.34-2.67) and long-term (OR 1.87; 95% CI 1.25-2.79) absenteeism. After adjusting for sex, age, and contract type (Model 1), job satisfaction remained associated with both shortand long-term absenteeism (Table 3).

**Table 3:** Unadjusted and adjusted models (multinomial regression) of the association between low job satisfaction and short-term (1-7 days) and long-term (≥8 days) absenteeism in public school teachers, Londrina, PR, Brazil, 2012-2013

Analysis models	Absenteeism			
	Short term		Long term	
	OR (95% CI)	P-value	OR (95% CI)	p-value
Unadjusted	1.89 (1.34-2.67)	<0.001	1.87 (1.25-2.79)	0.002
Model 1^*^	1.84 (1.30-2.60)	0.001	1.86 (1.24-2.79)	0.003
Model 2†	1.60 (1.12-2.30)	0.011	1.49 (0.97-2.28)	0.069

OR = odds ratio.

* Adjusted for sex, age, and contract type.

† Adjusted for sex, age, type of contract, reported chronic pain, reported chronic illness, physical work capacity, mental work capacity.

After all adjustments (Model 2), job satisfaction was only associated with short-term absenteeism (Table 3). Younger age was also associated with short-term absenteeism: 23-35 years old (OR 1.89; 95% CI 1.23-2.90); 36-46 years old (OR 1.63; 95% CI 1.11-2.40). There was also an association between long-term absenteeism, permanent contract type (OR 1.83; 95% CI 1.12-2.98) and chronic pain (OR 1.55; 95% CI 1.05-2.27). Teachers who reported chronic disease had a risk of short-term (OR 2.07; 95% CI 1.32-3.25) and long-term (OR 4.13; 95% CI 1.91-8.91) absenteeism. According to the confounder matrix, the sensitivity of the final model reached 76.9%, with 52.3% of the cases correctly classified.

## DISCUSSION

Lower job satisfaction remained associated with short-term absenteeism in all adjusted models, even though a lower OR was detected in the model that included health conditions. Statistical significance for long-term absenteeism was lost in the final model, which included health variables. Therefore, it can be assumed that pain and/or chronic disease is a more important factor in long-term absenteeism than lower job satisfaction. However, job dissatisfaction can be considered an important predictor of short-term absenteeism.

Although studies assessing the relationship between job satisfaction and absenteeism among teachers could not be found in the databases (SciELO, LILACS, PubMed, etc.), studies in other worker populations have found a positive association between lower job satisfaction and absenteeism.^9,10,17,19,24^ A survey of 874 workers at a public hospital in Chile^10^ found that job dissatisfaction was an important predictor of absenteeism, even after adjusting for sex, age, hierarchical level, and the hospital’s psychological climate. Likewise, in a case-control study with Brazilian oil industry workers, Oenning et al.^24^ found that those who reported job dissatisfaction took the most sick leave. Thus, although the present study evaluated a population of teachers, its results resonate with those found in other populations, demonstrating the need for further research into job satisfaction levels among elementary and secondary school teachers to guide attempts to reduce dissatisfaction and, consequently, absenteeism rates (especially short-term absenteeism) and related losses.

It was also observed that younger teachers had a higher risk of short-term absenteeism. Wargo-Sugleris et al.^25^ found that the relationships between job satisfaction and general well-being are stronger for older workers, and thus it is expected that older workers, when healthy, will be absent less often. Furthermore, due to their professional experience, older teachers generally have a greater ability to deal with conflict and may have lower stress levels and greater resilience.^26^

Chronic disease was associated with both shortand long-term absenteeism, while chronic pain was associated with long-term absenteeism. Impaired teacher health may result from the physical and emotional exhaustion experienced at work, which can increase the risk of health problems and lead to unplanned absences. Dos Santos & Marques^27^ point out that poor working conditions and the exhaustion they produce are major precursors to absenteeism among primary and secondary school teachers.

Teachers with permanent contracts had a higher risk of long-term absenteeism. This may be due, in the first place, to the greater difficulty that temporary teachers have in obtaining long-term medical leaves, given that, after the 15th day of leave, their only recourse is for social security sickness benefits, which can only be obtained through a complex a bureaucratic process. In the second place, permanent teachers are usually older (median 11 years) than their temporary peers.^28^ Although age (in tertiles) was included in the adjusted analyses, it is associated with chronic disease and other health problems, which are ultimately associated with long-term absenteeism.

A total of 50.4% of the teachers reported absenteeism in this study. This was similar to the results of the 2015-2016 Educatel Brasil Study, which found that 53.3% of more than 6000 teachers from all regions of Brazil reported absenteeism due to illness.^7^ However, other studies have found lower levels of absenteeism.^29,30^ These studies identified absenteeism by directly verifying the teachers’ presence at school during 12 months of data collection, the same period used in our study. The high rate of absenteeism in the present study may be due to lower general well-being among the teachers in our sample. As a result, workers tend to resort to absences, temporary leaves, and even unpaid leaves to escape a dissatisfying and fatiguing work environment.^31^

One limitation of our study was that the job satisfaction scale was self-applied, which could have led to interpretation errors by the respondents. Furthermore, asking teachers about work absences over such a long period (the previous 12 months) could have led to memory bias. However, some measures were taken to improve the study’s internal validity: conducting a pilot study, training interviewers and preparing a support manual, conducting interviews in a private environment, and double data entry. Nevertheless, the Cronbach coefficient (alpha = 0.943) for the job satisfaction scale indicated high reliability.

The results of the present study indicate that strategies to increase job satisfaction among teachers may help reduce absenteeism, especially short-term absenteeism, as well as prevent more serious outcomes, such as abandoning the profession. Initiatives to improve the general working conditions of teachers should prioritize the lowest rated dimensions, ie, by increasing salaries, reducing work volume, and expanding opportunities for professional growth.

## CONCLUSIONS

In summary, lower job satisfaction was associated with short-term absenteeism, regardless of sex, age, employment type, chronic pain, chronic illness, or physical or mental work capacity. Therefore, the results support the hypothesis that lower job satisfaction is a predictive factor for absenteeism.
